# Supplementary material for: Essentiality of Plasmodium falciparum plasmepsin V
Source: PLoS One. 2018 Dec 5;13(12):e0207621. doi: 10.1371/journal.pone.0207621 (PMC6281190; doi:10.1371/journal.pone.0207621)
Supplement: S1 Table — (PDF) [file pone.0207621.s001.pdf]

S1 Table. Oligonucleotide primer sequences used in this study

**Primer name**	**Sequence (5´ - 3´)**
PA-AflII-int_F	GCCTTCTTGACGAGTTCTTCTAACTCGAGCTTAAGGTAAATAAAAAAAATAATATAC
PB-NheI-int_R	CACATGTTAATAAACTTCCTCTTCCTTCTCCGCTAGCATCTAAAAGAATAT
PC-T2A_GFP_F	GTTTATTAACATGTGGAGATGTAGAAGAAAATCCAGGACCAATGAGTAAAG
PD-XhoI_GFP_R	CTGCCATATCCCTCGAGTTATTTGTAGAGCTCATCC
P1.1-BglII_PMV_F-mod	TAACAGACTTAGGAGGAGATCTTTTTTAAGGAAAGAAAATTTTTTTATATTG
P2-Int_PMV_R	TTGTATATTATTTTTTTTATTTACCTTTACAACCATTACAC
P3.1-BglII_5´UTR_F-mod	TAACAGACTTAGGAGGAGATCTCCGAAAAGACATTAATTTGTGTAAC
P4-PMV_int_F	GATACAGGTTCATCTTCGTTAAGTTTCCCGTGTAATGGTTGTAAAGGTAAATAAAAAAAA
P5-T2A_HA_R	AAACTTCCTCTTCCTTCTCCGTCGACAGCGTAATCTG
P6-5´UTR_Screen_F	CCTTATAAAGAATAAAATATGGTATGATAAAAGCTACGC
P7-T2A_Screen_R	CATCGTATGGGTAGGTAGATTCCTGGATCG
P8-GFP_screen_R	GGTCCTGGATTTTCTTCTACATCTCCACATG
P9-PMVmut_R	GGTTATACGGTTTTTCCATGTGTATGCCAC
P10-synPMV_h_F	GAGTTCTTCTAACTCGAGCTTAAGG
P11-synPMV_h_R	CTTCCTCTTCCTTCTCCGCTAGCAGCGTAATCTGGAAC
P12-A178C_R	GTAAATCTGTGAACCTTCACAATAAGACTGCAAATACTC
P13-A178C_F	GAGTATTTGCAGTCTTATTGTGAAGGTTCACAGATTTAC
P14-A365D_R	GAGTAAAAGTGCTACCAGAATCTACAAGCATGTCGAG
P15-A365D_F	CTCGACATGCTTGTAGATTCTGGTAGCACTTTTACTC
P16-EcoRV-PMV-F	TGTACAAGATATCAAAAGTTTAGAAAACCTACCAAATTTATATATTAC
P17-EcoRV-PMV-R	ATTTCAAGATATCCTATGTTGATTCCTGTATGGGAG
P18-sgPMV-1F	ATTGTTAGATATGTTAGTAGATTC
P19-sgPMV-1R	AAACGAATCTACTAACATATCTAA
P20-sgPMV-2F	ATTGTAGTTGATGGAGTACAATGT
P21-sgPMV-2R	AAACACATTGTACTCCATCAACTA
P22-PMV-endo-R	CCCTTCACAATACGATTGAAGATATTCAC
P23-rcPMV-5´integr-R	GTAGATGTTGGTCATGTCGTGGATGCAC
P24-GFP-3´integr-F	GTATACATCATGGCAGACAAACAAAAG
P25-3´UTR-PMV-R	GGGACAAATAAGGTCTTGCGTTGACCAC
Deu119_PreHR-pT2A_cKO_F	ACAGCTATGACCATGATTACGC
Deu196_NPT_seq_F	GATTGCACGCAGGTTCTCCGGCCG
Deu197_NPT_seq_R	CGGCCGGAGAACCTGCGTGCAATC
Deu198_GFP_start_seq_R	CCTCTCCACTCAGAAAATTTGTGCC
Deu199_GFP_end_seq_R	CAACATACACATTTTTACAGTTATAAATACAATCAATTG
Deu216_NPT_seq_F2	CATCGAGCGAGCACGTACT

* Restriction sites used in primers are underlined.
