# Supplementary material for: Essentiality of Plasmodium falciparum plasmepsin V
Source: PLoS One. 2018 Dec 5;13(12):e0207621. doi: 10.1371/journal.pone.0207621 (PMC6281190; doi:10.1371/journal.pone.0207621)
Supplement: S1 Fig — The loxPint and gfp fragments were amplified from pT2A-DDI-1cKO-complement. Overlapping PCR was performed to join the two fragments. The AflII and NheI restriction sites were introduced to facilitate further cloning. pT2A-DDI-1cKO was digested with XhoI. The digested fragment was ligated with loxPint-modified GFP fragment at the XhoI site. (PDF) [file pone.0207621.s002.pdf]

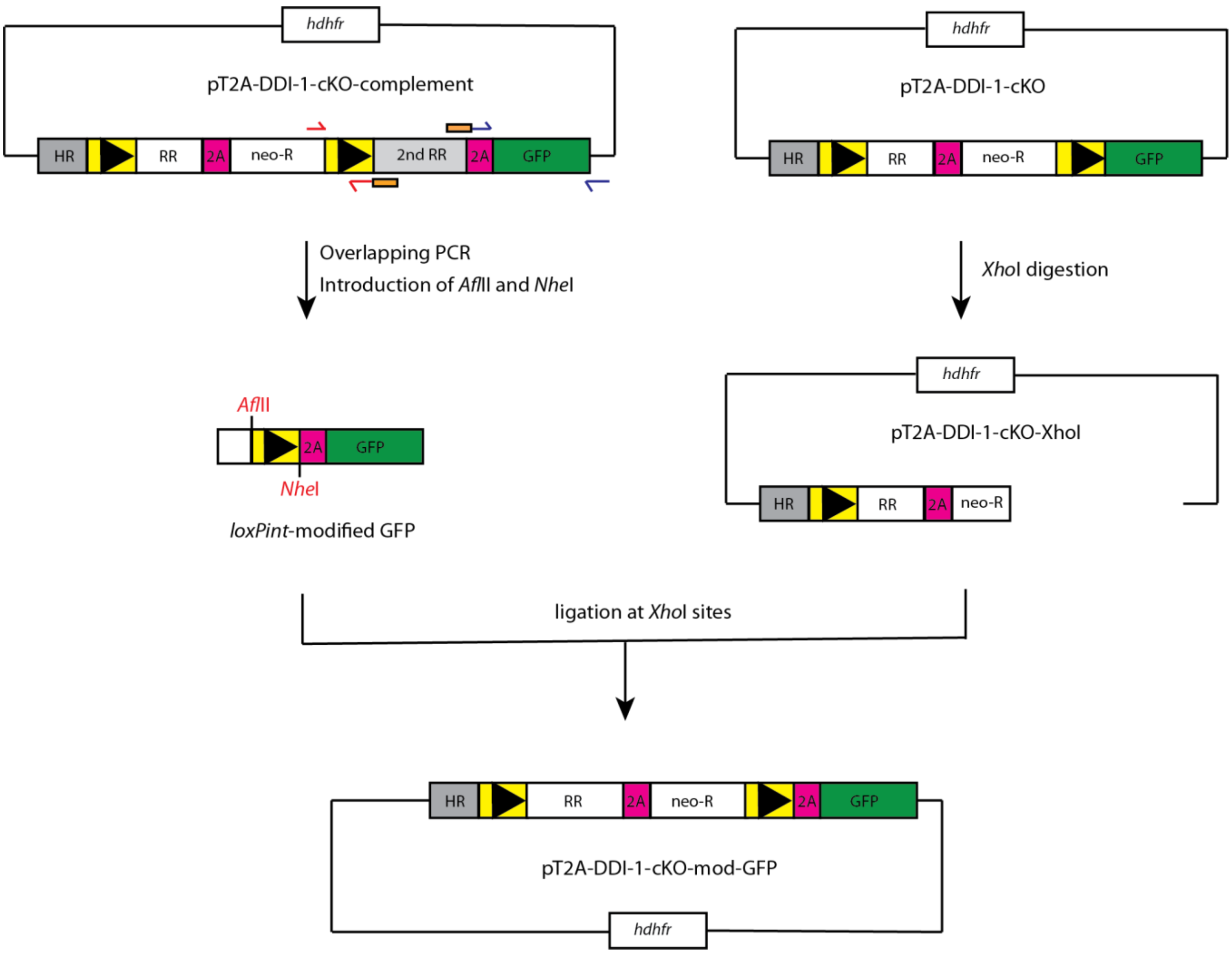

S1 Fig. Construction of pT2A-DDI-1-cKO modified GFP. The *loxPint* and *gfp* fragments were amplified from pT2A-DDI-1cKO-complement. Overlapping PCR was performed to join the two fragments. The *Afl*II and *Nhe*I restriction sites were introduced to facilitate further cloning. pT2A-DDI-1cKO was digested with *Xho*I. The digested fragment was ligated with *loxPint*-modified GFP fragment at the *Xho*I site.
